# Acid Gradient across Plasma Membrane Can Drive Phosphate Bond Synthesis in Cancer Cells: Acidic Tumor Milieu as a Potential Energy Source

**DOI:** 10.1371/journal.pone.0124070

**Published:** 2015-04-15

**Authors:** Gautam Dhar, Suvajit Sen, Gautam Chaudhuri

**Affiliations:** 1 Department of Obstetrics and Gynecology, David Geffen School of Medicine at UCLA, 10833 Le Conte Avenue, Los Angeles, CA, 90095-1740, United States of America; 2 Department of Molecular and Medical Pharmacology, David Geffen School of Medicine at UCLA, 10833 Le Conte Avenue, Los Angeles, CA, 90095-1740, United States of America; 3 Jonsson Comprehensive Cancer Center, Los Angeles, CA, United States of America; University Paris South, FRANCE

## Abstract

Aggressive cancers exhibit an efficient conversion of high amounts of glucose to lactate accompanied by acid secretion, a phenomenon popularly known as the Warburg effect. The acidic microenvironment and the alkaline cytosol create a proton-gradient (acid gradient) across the plasma membrane that represents proton-motive energy. Increasing experimental data from physiological relevant models suggest that acid gradient stimulates tumor proliferation, and can also support its energy needs. However, direct biochemical evidence linking extracellular acid gradient to generation of intracellular ATP are missing. In this work, we demonstrate that cancer cells can synthesize significant amounts of phosphate-bonds from phosphate in response to acid gradient across plasma membrane. The noted phenomenon exists in absence of glycolysis and mitochondrial ATP synthesis, and is unique to cancer. Biochemical assays using viable cancer cells, and purified plasma membrane vesicles utilizing radioactive phosphate, confirmed phosphate-bond synthesis from free phosphate (P_i_), and also localization of this activity to the plasma membrane. In addition to ATP, predominant formation of pyrophosphate (PP_i_) from P_i_ was also observed when plasma membrane vesicles from cancer cells were subjected to trans-membrane acid gradient. Cancer cytosols were found capable of converting PP_i_ to ATP, and also stimulate ATP synthesis from P_i_ from the vesicles. Acid gradient created through glucose metabolism by cancer cells, as observed in tumors, also proved critical for phosphate-bond synthesis. In brief, these observations reveal a role of acidic tumor milieu as a potential energy source and may offer a novel therapeutic target.

## Introduction

Warburg effect is a metabolic hallmark of most aggressive cancer cells whereby most of the glucose is converted to lactate in presence of oxygen [1, 2]. The aerobic glycolysis is accompanied by acidification of the tumor microenvironment [3, 4] that confers selective growth advantage to cancer cells by metabolic reprogramming [5–7], increased invasiveness [8–10] and regulation of cell cycle [11]. Aggressive cancer cells require ATP to generate enough building blocks like proteins, lipids and DNA for proliferation. Conversion of glucose to lactate produces only 2 molecules of ATP as opposed to 38 molecules when coupled to oxidative phosphorylation [12]. Therefore, even though aerobic glycolysis and extracellular acidification confers selective growth advantage to cancer as discussed above, as to how the cancer cells meet its energy demand under this condition remains a paradox.

Acid gradient or proton-motive force across membranes is a source of energy which is used by the mitochondria, chloroplast and the microbial world to synthesize phosphate bonds [12–16]. First proposed by Peter Mitchell in his famous chemo-osmotic theory [17], acid gradient across membrane remained the most widely utilized method in living systems to store the energy of metabolic fuels as electrochemical potential energy. The cellular machinery subsequently utilizes this energy to drive the synthesis of high-energy phosphate bonds in the form of ATP and other high-energy molecules for utilization in cellular processes. [13, 16].

It is interesting to note that even though the extracellular pH of cancer cells is acidic, the intracellular pH is more alkaline when compared to normal cells [3, 18, 19]. The alkaline intracellular pH and acidic extracellular pH with the plasma membrane in between represents a substantial pool of proton-motive energy. Tumors being able to utilize this stored potential energy to drive high-energy phosphate-bond synthesis in the cells remain a possibility. There is strong physiological evidence for the importance of acid gradient in cancer that are already established by other investigators. It was observed that increasing the extracellular pH by injection of alkaline buffers into the tumor environment reduced the tumor size and also inhibited metastasis [20, 21]. Moreover, cancer cells exhibit enhanced proliferation and invasiveness in acidic external environment [8–10, 22]. This in-vivo physiological evidence indicate that acid gradient, independent of the mode of acid secretion, stimulate proliferation of cancer cells and are also supportive of their energy needs. However, direct biochemical evidence linking extracellular acid gradient to generation of intracellular ATP have been lacking, and this is the focus of the present work. It is challenging to confirm ATP synthesis in response to acid gradient with certainty while eliminating contributions from glycolysis or mitochondria in an in-vivo system. Inhibition of glycolysis or mitochondria in-vivo would be lethal. However, this can be investigated utilizing cultured cells and purified plasma membrane vesicles. Radioactive phosphate can be used to confirm whether the new phosphate bonds are formed from free phosphate and not by phosphate bond exchange. The work presented here confirms that cancer cells can synthesize significant amounts of phosphate bonds from phosphate in response to acid gradient across the plasma membrane.

## Materials and Methods

### Materials

Cell lines used were obtained from ATCC and were cultured as directed. Cells were harvested when 80–85% confluent by scrapping with cold PBS containing 10% FBS, without treatment with trypsin, to preserve the surface proteins. ^32^P_i_ were obtained from Perkin Elmer and was treated with shrimp alkaline phosphatase followed by heat inactivation to remove contaminating PP_i_. All other chemicals unless otherwise mentioned were purchased from Sigma Chemicals.

### Assay for the amounts of ATP in cells in response to acid gradient

Cell suspensions (2–3 million cells/ml) in PBS or bis-tris buffer, containing 10% FBS, and brought to steady state by incubating at 37°C for 20–30 minutes, were acidified either by adding 4 volumes of reaction buffer (20 mM bis-tris, 150 mM NaCl, 5 mM KCl, 5 mM MgCl_2_ or by adding small aliquots of dilute HCl. The reaction was quenched by vortexing with chloroform. The aqueous layer was used to measure ATP using the Luciferase luminescence assay kit (Promega).

### Determination of order of reaction (n)

Slope of the line of log (A/A_o—_1) or log (A—A_o_) against pH gives the order of reaction. A_o_ and A are the amounts of ATP before and after addition of acid respectively. Details of the mathematical model are given in the supplement S1 Fig.

### Loading cells with ^32^P_i_ and response to acid

Approximately 10–12 million cells were harvested from culture plates by scrapping in cold PBS-20% FBS. They were then washed with cell suspension buffer SB which is 50 mM Hepes-MES (1:1) pH 7.6, 100 mM NaCl, 2 mM KCl, 2mM MgCl_2_, 0.2mM CaCl_2_, 0.4 mM spermine (relatively fresh), 0.25 mg/ml BSA, and then suspended in 1 ml of the same buffer, incubated at 37°C for 30 minutes with occasional mixing. The cell pellet was recovered and suspended in fresh 500 μl buffer SB and incubated at 37°C for 5 minutes. To this was added 5 μM oligomycin and 1 μM atractyloside and kept on ice for 15 minutes. Ouabain (0.2 mM) was added and incubated on ice for another 15 minutes. Local acidification due to settling of the cells was avoided in all the steps by gently inverting the tubes occasionally. After incubation at 37°C for 5 minutes, a mixture of cold sodium phosphate pH 7.5 and radioactive phosphate (^32^P_i_) was added such that the final phosphate concentration was 1 mM with approximately 0.20 mCi/ml of radioactivity. This was then incubated in cold for 45 minutes with gentle rotation. The cell pellet was recovered by centrifugation and washed with 500 μl of wash buffer (WB) which was buffer SB containing 1 mM sodium phosphate, 0.2 μM bafilomycin in addition to oligomycin, atractyloside and ouabain as before. The cells were suspended in WB to a density of 10 million cells/ml and aliquots of approximately 175 μl were made in separate 1.5 ml tubes. After incubation for 1 minute at 37°C, the cell suspensions were acidified to the desired pH by adding small aliquots of dilute HCl and incubated for 45 seconds to 1 minute, after which the cells were pelleted by centrifuging at low speed, and the supernatant was removed. To the pellet was added 75 μl of chloroform and vortexed. The pellet should get suspended in chloroform for effective lysis of the cell. From the addition of acid up to the point when chloroform was added was considered as the incubation time in acid and was typically around 90 seconds to 2 minutes. 50 μl of extraction buffer (EB) which was 1 mM potassium phosphate pH 6.5, 0.2 mM EDTA, was added, vortexed and then kept in vibrator for 10–15 minutes. This was then centrifuged and the aqueous layer was gently taken out and kept at -80°C. For analysis of the sample, approximately 20 μl of the aqueous extract were dried by speed-vac and analyzed by TLC. Aliquots were also used to estimate the absolute amount of ATP using luciferase luminescence assay kit (Promega).

### Enzymatic characterization of the products from ^32^P_i_ loaded cells

The aqueous extract from the acidification of cells were diluted with 4 volumes of buffer containing 10 mM Tris pH 8.0, 0.2mM EDTA, 1 mM MgCl_2_. 10 μl of this reaction mixture was then treated with different enzymes (1 unit) individually or in combination along with added substrates. A total of 8 different reactions were done. Each reaction had a specific aim in the identification of the nature of the nucleotides as detailed below. The disappearance of a band during a characteristic reaction indicated its identity. 1. Alkaline phosphatase (P-ase, Promega)—removes terminal phosphate groups with monoester linkage. So it can indicate if the bands have terminal phosphate bonds. 2. Inorganic pyrophosphatase (PP_i_-ase, from New England Biolab)—hydrolyses pyrophosphate (PP_i_). GTP migrates at the same place as PP_i_ in the TLC, so this reaction can differentiate between PP_i_ and GTP. 3. Phosphodiesterase (PDE)—cleaves the α-β phosphodiester linkage therefore it can generate PP_i_ from the NTPs and phosphate from NDPs. So bands of NDP and NTP disappeared with appearance of a band for PP_i_. 4. PDE followed by PP_i_-ase—PP_i_ generated from the reaction with PDE was cleaved by PP_i_-ase which confirmed that it is PP_i_ and not GTP. 5. Adenylate kinase (ADK) and 1 mM AMP—it converts ATP to ADP, so the ATP band diminished, while ADP band increased. GTP can also act as weak substrate for this reaction so GTP band also diminished. 6. Nucleotide diphospho kinase (NDK) and 1 mM ATP—NDK can transfer terminal phosphate from ATP to the NDPs, so ADP and GDP bands disappeared. 7. NDK and 1 mM ADP—ADP converted to ATP by phosphate transfer from GTP, so the GTP band disappeared.

### Preparation of plasma membrane

Plasma membrane was prepared by the usual method of swelling of cells in low salt buffer and rupturing by passing through a narrow 25 gauge needle. The buffers and timing were chosen to best preserve the activity. EDTA was not added during the purification. Typically 200 million cells were scrapped and harvested from culture plates with PBS-20% FBS and washed with 5 ml of 2.5 mM potassium phosphate buffer pH 7.5 and suspended in 10 ml buffer A which was 10 mM Hepes-MES (1:1), 100 mM NaCl, 2 mM KCl at pH 7.0. To this was added 0.1 mM PMSF and 2% FBS and kept on ice for 20 minutes after which sodium phosphate pH 7.5 was added to 1 mM. The cell suspension was lysed by 20 full strokes with 10 ml syringe fitted with 25-gauge needle while still keeping on ice. The lysed suspension was centrifuged at 1000xg for 5 minutes to remove intact cells and nucleus and then at 5000xg for 15 minutes to remove mitochondria. The relatively clear supernatant from this step was then centrifuged at 75000xg for 40 minutes in an ultracentrifuge. The supernatant was drained out completely and the pellets were pooled together and washed with buffer B which was buffer A containing 1 mM sodium phosphate and 0.5 mg/ml ultra-pure BSA (Invitrogen). The pellets were then suspended in 300 μl buffer A containing 0.5 mg/ml ultra-pure BSA and kept as 75 μl aliquots at -80°C. Multiple freeze-thaws greatly affected the activity of the enzymes. To preserve the activity of the enzyme, the pellets formed during centrifugation were always suspended by gentle pipetting and strong vortexing was avoided at all steps. All operations were done in cold or on ice.

#### Protein concentration in the membrane preparation

To determine the protein concentrations, the membrane preparations were done in absence of BSA. They were dissolved in 0.5% SDS and amount of protein was estimated using BCA protein assay kit (from Pierce). Typically, the protein concentrations were in the range of about 0.5 mg/ml. Membranes equivalent to approximately 12–15 μg protein per reaction were used as starting membrane concentration during vesicle preparation.

#### Membrane purity and western blot

Immunoblot analysis utilizing standard techniques were performed to establish the purity of the plasma membrane fractions. The membrane pellets were dissolved by heating in 1% SDS at 65°C for 10 minutes. Whole cell extracts heated in 1% SDS at 65°C for 10 minutes was used as control. Antibodies against Na-K ATPase (ab7671, Abcam Cambridge) and E Cadherin (ab15148, Abcam, Cambridge) were used to determine the enrichment of plasma membrane proteins. Antibodies against VDAC (ab34726, Cambridge, MA) and COXIV (ab124538, Abcam, Cambridge) were used to rule out contaminations from mitochondrial membrane fractions. Antibodies against GAPDH (ab8245, Abcam, Cambridge) were utilized to rule out cytoplasmic contamination.

### Preparation of rightside-out plasma membrane vesicles and acid response

The method stated here is a prototype and most of the time it was scaled according to the need of the experiment. Typically 150 μl plasma membrane preparation was diluted to 300 μl (depends on density of the membrane preparation) with buffer A containing 0.5 mg/ml BSA and then suspended well by applying 10 strokes with 1 ml syringe fitted with 25 gauge needle. The final volume was checked and if necessary was adjusted to 300 μl with the same buffer. In the following steps, the ingredients were strictly added in the order mentioned considering a final volume of 400 μl. The ingredients were added sequentially to a final concentration as indicated: Hepes-MES (1:1) pH 7.0 to 50 mM, KCl to 20 mM, sodium phosphate to 1 mM, ouabain to 0.2 mM, oligomycin 10 μM, bafilomycin to 200 nM, atractyloside to 1 μM and BSA to 1 mg/ml. ADP or other ingredients that needed to be packed into the vesicle were also added as necessary. The pH was then adjusted to 7.6 with 1 N sodium hydroxide and confirmed by pH electrode. The volume was checked and if necessary was adjusted with water to get a final volume of 400 μl after addition of radioactivity. This was then subjected to 10 strokes with 1 ml syringe fitted with 25 gauge needle before adding radioactivity. 0.4 mCi/ml of ^32^P_i_ was then added and kept on ice for 20 minutes after which 10 strokes were applied with syringe. The vesicles were now sealed by the following sequence of steps. Relatively fresh spermine solution was added to 1.6 mM, applied 10 strokes with syringe, then MgCl_2_ was added to 4 mM, applied another 10 strokes with syringe and kept on ice for 20 minutes. To this was added valinomycin to 10 μM, kept on ice for 10 minutes followed by CaCl_2_ to 0.4 mM and incubated on ice for another 10 minutes. Magnesium over 6 mM, spermine over 1 mM (final after dilution) and calcium over 1 mM inhibited activity so excess addition was always avoided.

#### Binding to ConA beads

300 μl ConA beads (Rad/GE Healthcare) were washed two times in 1 ml of buffer containing 10 mM Hepes-MES (1:1) pH 7.0, 100 mM NaCl, 1 mM CaCl_2_, 0.25 mM BSA (no MnCl_2_ was added). After the last wash all the liquid was aspirated out by pipetting with fine gel loading tips (that do not allow beads to get in) by dipping it deep into the beads. This was suspended in 2 ml tube with 3 volumes of dilution buffer (DB) with respect to the vesicle preparation, which in this case would be 1.2 ml. The composition of the DB was 50 mM Hepes-MES (1:1) pH 7.6, 100 mM NaCl, 1 mM sodium phosphate, 4 mM MgCl_2_, 0.4 mM CaCl_2_. To the bead suspension was added 400 μl of sealed vesicle preparation (from above). Dilution of potassium at this stage helps in the binding to ConA beads, which are usually inhibited at high potassium. The tube was sealed securely and rotated very gently in cold for 50 minutes to allow binding without harming the membrane. The beads were washed 3 times with 3 volumes of ice cold wash buffer (WB) by centrifuging at 1500 rpm 30 seconds. The wash buffer contained 50 mM Hepes-MES (1:1) pH 7.6, 100 mM NaCl, 1 mM sodium phosphate, 2 mM KCl, 4 mM MgCl_2_, 0.4 mM CaCl_2_, 0.4 mM spermine, 0.25 mg/ml ultra-pure BSA, 0.1mM ouabain, 2.5 μM oligomycin, 100 nM bafilomycin, 1 μM atractyloside. Each time all liquids were aspirated out from the bed with fine gel loading tips by dipping it deep down into the beads. Efficiency of wash was checked by reduction of radioactivity of the beads between successive wash using Geiger counter. The beads were then suspended in about 1 ml of wash buffer, aliquoted evenly to about 6 tubes (195 μl suspension) and immediately used for vesicle assay as detailed below.

#### Bead bound vesicle assay

The bead suspension was incubated at 37°C for 1 minute and to it added measured amounts of 1 N HCl to shift the pH to desired values. After desired time interval (5 seconds to 2 minutes), it was centrifuged for 15 seconds at 1000 rpm and the supernatant was quickly removed with fine gel loading tips by dipping it into the beads. 75 μl of chloroform-2.5% methanol mixture was then added to the beads and vortexed. To this was added 50 μl of extraction buffer EB (1 mM potassium phosphate pH 6.5, 0.2 mM EDTA). The mixture was then kept in vibrator for 30 minutes, centrifuged at 8000 rpm for 10 minutes and the top aqueous layer was carefully taken out avoiding chloroform using fine gel loading tips. The beads were re-extracted with another 25 μl of EB, by vortexing for 5 minutes and centrifuging as before. The aqueous layers from this two steps were pooled together and centrifuged at 10000 rpm for 1 minute to precipitate any beads and the clear aqueous layer were taken out. This was then kept at -80°C. For analysis of the sample, approximately 25–30 μl of the aqueous extract were dried by speed-vac and analyzed in TLC.

### Inside-out vesicle (IOV) assay

Membrane vesicle were taken in buffer containing 10 mM Hepes-MES-Acetate (1.5:1:1) pH 6.34, 2 mM KCl, 150 mM NaCl, 10 μM oligomycin and sealed with 6 mM MgCl_2_. Added 10 μM valinomycin followed by one-fourth volume of the above buffer but containing 150 mM KCl instead of NaCl. ^32^P_i_ (0.2–0.5 mM, 0.3 mCi/ml) and ADP (25 μM, when necessary) were then added. After brief alkalization for 90 seconds by adding alkali, 20 μM of pyrophosphate, and/or 20 μM of ATP were added to dilute (protect) the radioactive products and vortexed with chloroform (containing 2% methanol). The aqueous layer was analysed by TLC.

### Preparation of cytosolic extract

Cells (100 million/ml) were allowed to swell in 2 mM K_2_HPO_4_ pH 7.4 for 5 minutes after which the buffer was adjusted to 10 mM and NaCl to 100 mM. To this was added 1 mM diethylpyrocarbonate, 50 μM vanadate, 50 μM pyrophosphate and lysed by passing through 25 guage needle. This was centrifuged twice at 13,000xg for 15 minutes and then either centrifuged at 74000xg for 40 minutes or sequentially passed through 0.65 μm, 0.22 μm and 0.11 μm membrane column to remove membranes. Protein content was approximately 3 mg/ml.

### IOV and cytosolic extract combination assay

Cytosol (5 ug protein) was added to IOV mixture containing ^32^P_i_ (0.2 mM, 0.3 mCi/ml) and ADP (100 μM) and quickly alkalinized for 10 seconds. The reaction was quenched with 2 mM diethylpyrocarbonate and 1/1000^th^ phosphatase inhibitor cocktail B (Santa Cruz Biotech) and vortexing with chloroform (containing 2% methanol). The aqueous layer was analyzed by TLC.

### Assay for conversion of ^32^PP_i_ to ATP by cytosolic extract

Cytosolic extracts (2 μg protein) was used per 50 μl reaction that contained 10 mM Tris pH 7.5, 1 mM NaH_2_PO_4_, 100 mM NaCl, ^32^PP_i_ (200 μM, 30 μCi/ml), 1 mM MgCl_2_ and 250 μM ADP. The reactions were quenched with 1 mM NHS-biotin (Pierce) and then analyzed on TLC.

### Thin Layer Chromatography (TLC)

PEI-cellulose TLC plates of 250 μm thickness (from J.T. Baker Inc.) were used. The spots were developed with 0.75 M KH_2_PO_4_, pH 3.5.

### Statistics

All results shown as averages are presented as mean ± 2.SD (SD: standard deviation) from three or more independent experiments. Unless otherwise noted, p values were calculated using Student’s two-tailed test: (p < 0.05 was considered as significant).

## Results

### Extracellular acid gradient is critical for high ATP levels during aerobic glycolysis

**Fig 1:**
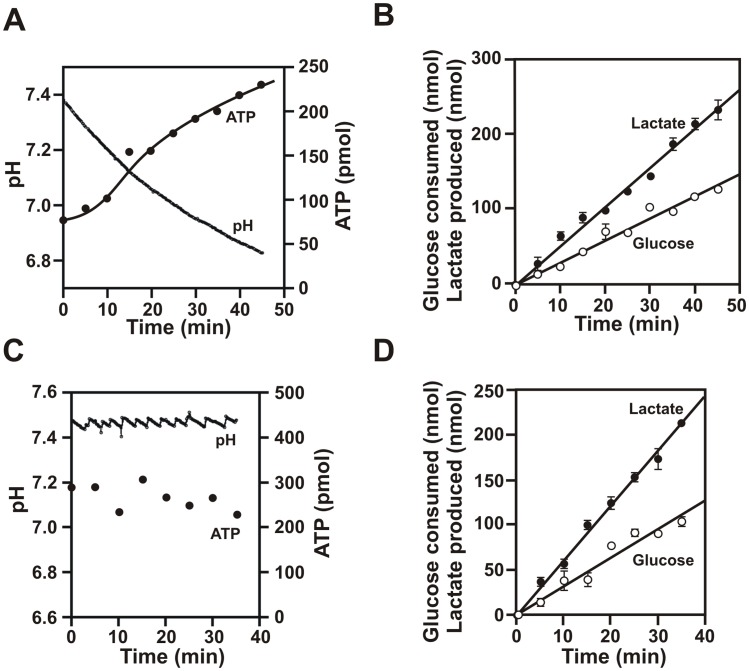
Cancer cells require extracellular acidic pH to maintain high levels of ATP. To MDA-MB-231 cell suspensions in PBS-10% FBS containing 10 μM oligomycin was added 0.1% glucose under gentle mechanical stirring. The pH of the medium (extracellular pH) was monitored in real time while ATP, glucose and lactate were measured (per million cells) from aliquots taken out at indicated time points (error bars = 2.s.d., n = 3). (A) Steady state ATP and acid extrusion (pH) during glycolysis. The ATP values increased continuously relative to the starting value with gradual decrease in the pH of the external medium. (B) Glucose consumed (open circle) and lactate produced (solid circle) with time for the experiment in panel A. (C) Steady state ATP upon continuous neutralization of acid. The secreted acid was continuously neutralized by adding small aliquots of alkali. The ATP values remained almost unchanged relative to the starting value under this condition. (D) Glucose consumed (open circle) and lactate produced (solid circle) for the experiment in panel C. These experiments were repeated three times.

During aerobic glycolysis cancer cells consume glucose to form lactate with simultaneous extrusion of acid which is reflected by the lowering of pH of the external medium [23]. Initially, we wanted to investigate the critical role of acid gradient in maintaining ATP levels during aerobic glycolysis. We designed an experiment to simultaneously measure the levels of ATP, lactate, glucose and the pH of the external medium in real time during aerobic glycolysis of highly glycolytic breast cancer cells, MDA-MB-231[24]. We also added 10 μM oligomycin, a bonafide mitochondrial ATP synthesis inhibitor, to eliminate any contribution of mitochondrial ATP synthesis. Glycolysis was initiated by adding 0.1% glucose. Aliquots from the cell suspensions were taken out every 5 minutes, added into chloroform and immediately vortexed for effective lysis of the cells and inactivation of the enzymes. The aqueous layer was used for the estimation of water-soluble metabolites like ATP, glucose and lactate. We observed that as glucose was consumed to form lactate, there was a steady decrease in pH with time accompanied by simultaneous increase in the steady state levels of ATP in the cells relative to the starting value (Fig 1A). The cells consumed glucose at a rate of 3.08 nmol/min/million-cells (s.d. = ±0.24, 7 data sets) and produced lactate at a rate of 4.95 nmol/min/million-cells (s.d. = ±0.46, 7 data sets) (Fig 1B). This amounts to almost 80.7% (s.d. = ±8.78, 7 data sets) conversion of glucose to lactate under this condition which are similar to that seen by other investigators [25, 26]. On the other hand, when the extruded acid was constantly neutralized by adding small aliquots of alkali so that the extracellular pH remained constant at around 7.4, levels of ATP did not increase with time relative to the starting value (Fig 1C). However, the rates of glucose consumption (3.46 nmol/min/million-cells, s.d. = ±0.17, 3 data sets) and lactate production (5.95 nmol/min/million-cells, s.d. = ±0.50, 3 data sets) exhibited no significant change (Fig 1D). This experiment indicated that the extracellular acid gradient plays a critical role even during aerobic glycolysis in maintaining high levels of ATP in cancer cells. This encouraged us to investigate if extracellular acid gradient alone can drive ATP synthesis in cancer cells in absence of any added glucose.

### Extracellular acid gradient alone is sufficient to drive ATP synthesis in cancer cells

**Fig 2:**
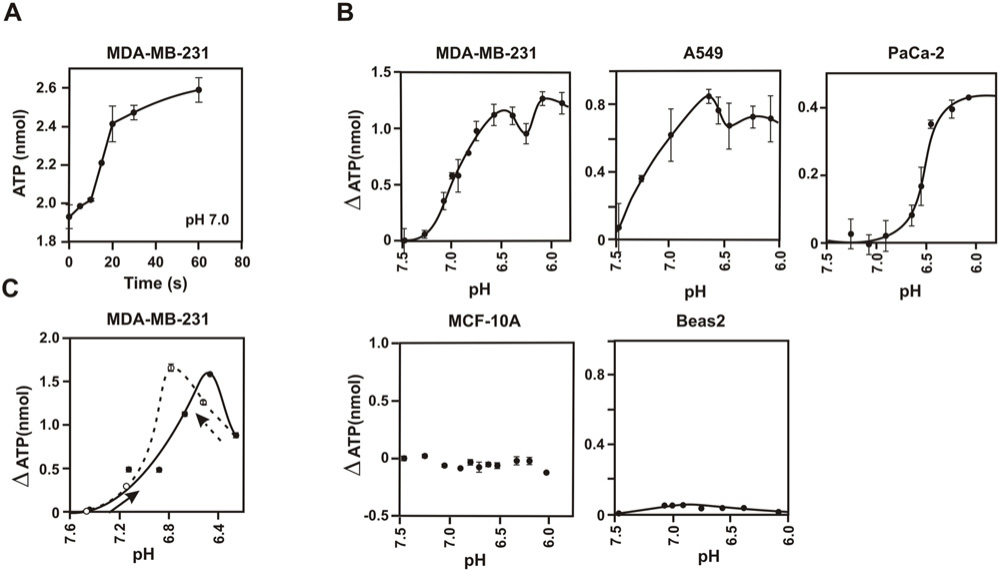
Cancer cells can produce ATP in response to extracellular acid alone. (A) Time kinetics of ATP production at pH 7.0. MDA-MB-231 (steady state), at pH 7.5 in glucose-free buffer, were acidified to pH 7.0 at 37°C for the indicated time, quenched with chloroform and the amounts of ATP were determined from the aqueous fraction. The ATP values are per million cells (error bars = 2.s.d., n = 3). (B) Increase in steady state levels of ATP with decrease in extracellular pH. Cell suspensions (steady state) at pH 7.5 in glucose-free buffer were acidified to the indicated pH for 2 minutes at 37°C and quenched with chloroform. The amounts of ATP in the cells were determined from the aqueous fraction. Increase in ATP per million cells are shown (error bars = 2.s.d., n = 3). The scales were kept similar for breast cells MDA-MB-231 and MCF-10A, and for lung cells A549 and Beas2 for comparison. The order of reaction (n) for this experiment using steady state equation (see methods) is shown in the supplement (S2 Fig). The average values of n (slope) from multiple such experiments are: MDA-MB 231, 0.89 (s.d. = ±0.07, 7 data sets); A549, 0.79 (s.d. = ±0.06, 4 data sets) and PaCa-2, 2.12 (s.d. = ±0.23, 6 data sets). (C) Reversible nature of acid gradient dependent ATP formation. MDA-MB-231 cell suspension (steady state) at pH 7.5 in glucose-free buffer was acidified in stepwise manner from pH 7.5 to 6.2 by adding small aliquots of acid under gentle stirring condition at 37°C. At each step the pH of the cell suspension was noted and aliquots were taken out after 2 minutes for determination of ATP (solid circle with solid line). After reaching pH 6.2, aliquots of alkali were added in a stepwise manner to bring the pH back to 7.5 (open circle and dotted line). The increase in ATP levels are per million cells (error bars = 2.s.d., n = 3).

We wanted to assess whether extracellular acid gradient alone is sufficient to drive ATP synthesis in cancer. The breast cancer cell line MDA-MB-231 in glucose-free buffer was used to standardize the assay and to understand the basic characteristics of the reaction. The cells were taken in glucose-free suspension buffer and kept at 37°C for 20 minutes. This was necessary to allow the cells to consume cytosolic glucose and to bring the basal levels of ATP to a steady state for the duration of the experiments. They were acidified and then lysed with chloroform after the indicated time (5 seconds to 2 minutes). ATP produced was estimated from the aqueous layer by luciferase assay. We observed that the ATP production in response to extracellular acid was fast and robust. ATP concentration in the cells increased rapidly within 20 seconds and reached a steady state by 1 minute upon shifting the pH from 7.5 to 7.0 (Fig 2A). The rate of ATP synthesis calculated from the first 20 seconds was approximately 1.5 nmoles/min/million-cells. The net increase of ATP levels at steady state was about 0.7 nmoles per million cells for this example. This is equivalent to an increase of 0.35 mM of ATP in the cells [27]. This indicates substantially robust synthesis of ATP considering that the steady state concentration of ATP in cells is around 1 mM. At pH 6.7, the rate of ATP synthesis was too fast to accurately monitor before it attains the steady state. We monitored the amount of ATP increase after 5 seconds of acidification and estimated an approximate rate of about 5.5 nmoles/min/million-cells (s.d. = ±1.3, 3 data sets). The cells retained their viability and integrity under these conditions as determined by trypan blue exclusion. Almost all the ATP produced (>98%) was found in the cell pellet indicating exclusive intracellular production of ATP. These experiments confirmed that extracellular acid gradient by itself is sufficient to drive ATP synthesis in cancer cells. Membrane rupture by freeze-thaw, mechanical homogenization or sonication also abolished this activity indicating that intact membrane was essential for this phenomenon.

Following these initial experiments, the effect of extracellular pH on the steady state levels of ATP was assessed at pHs' ranging from 6.0 to 7.5. We used a panel of aggressive cancer cell lines obtained from different tissue types, MDA-MB-231 (breast cancer), A549 (lung cancer) and MIA-PaCa-2 (pancreatic cancer, henceforth called PaCa-2), in order to determine the universality of the phenomenon. For comparison we utilized immortalized non-cancer cell lines MCF-10A (breast), Beas2 (lung) and also normal human mammary epithelial cell line HMEC (breast). We observed that the steady state levels of ATP in the cells started to rise at around pH 7.2 and continued till pH 6.0 as seen in the examples in Fig 2B, representing several fold increase in ATP levels. The pH of tumor environment ranges between 6.15 to 7.4 [3], which therefore appears to be compatible for the synthesis of ATP. Non-cancer cell lines MCF-10A and Beas2 gave only a negligible response (Fig 2B). Normal cell HMEC as well as mitochondria isolated from both MDA-MB-231 and MCF-10A did not exhibit any response. The fact that normal cells and immortalized cells failed to respond to acid gradient indicates that the traditional ATP synthesis pathways like glycolysis, oxidative phosphorylation or adenylate kinase reactions that are also present in these cell lines were not contributing towards this acid gradient driven ATP synthesis in cancer cells. ATP levels returned to their initial values upon stepwise reversal of pH (Fig 2C), suggesting that the steady state ATP levels in cancer cells is dependent on extracellular pH.

2-dexyglucose (2-DG) can inhibit glucose utilization by inhibiting hexokinase, a key enzyme of the glycolytic pathway [28]. Therefore, it can block ATP production from glycolysis as well as the mitochondria. We observed that even in the presence of 2 mM to 5 mM 2-DG, MDA-MB-231 could produce ATP in response to external acidification. At 2 mM 2-DG, 23% inhibition was observed (s.d. = ±2.9%, 3 data sets, p < 0.05). This fact, along with the inability of MCF-10A, HMEC or HUVEC to show any significant response in acid, further confirmed that ATP synthesis from glycolytic pathway or mitochondrial oxidative phosphorylation did not contribute towards the acid responsive ATP synthesis in cancer cells. Oligomycin, an inhibitor of mitochondrial ATP synthesis at the concentration range 1–5 μM [29], showed only 27% inhibition (s.d. = ±6.1%, 3 data sets, p< 0.05) of activity at a concentration of 10 μM. Bafilomycin, a potent inhibitor for the V-type ATPase at nanomolar range [30], showed only 25% inhibition (s.d. = ±7.2%, 3 data sets, p < 0.05) of activity, even at a concentration of 1 μM indicating that these chemicals does not effectively inhibit the activity.

In viable cells, ATP is constantly produced and consumed. The ATP measurements shown above (Fig 2B) represented the steady state levels of ATP. From the ATP vs pH curve in Fig 2B, simple mathematical model of steady state (S1 Fig) allowed us to calculate the relationship of rate of the ATP synthesis, [d[A]/dt)_H_], to the extracellular acid concentration, [H]. The rate is related to the acid concentration as (d[A]/dt)_H_ α [H]^n^ where n is the order of reaction. Slope of the line of log (A/A_o—_1) against pH gives the order of reaction (S1 Fig). A_o_ and A are the amounts of ATP before and after addition of acid respectively. The average value of n for MDA-MB-231 was 0.89 (s.d. = ±0.07, 7 data sets) and that for A549 was 0.79 (s.d. = ±0.06, 4 data sets) while the same for PaCa-2 was about 2.12 (s.d. = ±0.23, 6 data sets) (S2 Fig). Therefore, the rate of ATP synthesis changed almost linearly (first order) with extracellular acid concentration for MDA-MB-231 and A549 but changed as second order for PaCa-2.

### ATP (other nucleotides also) is synthesized from phosphate in response to acid gradient

The experiments discussed above indicated that ATP is synthesized in response to extracellular acid gradient in cancer cells. We wanted to confirm that the synthesis of ATP was due to the formation of new phosphate bonds from free phosphate and not from phosphate bond exchange reactions catalyzed by adenylate kinases (ADK) or nucleotide diphospho kinases (NDK). These classes of enzymes do not have the ability to add a free phosphate moiety [12]. We therefore utilized radioactive phosphate to monitor phosphate bond formation in response to acid gradient.

**Fig 3:**
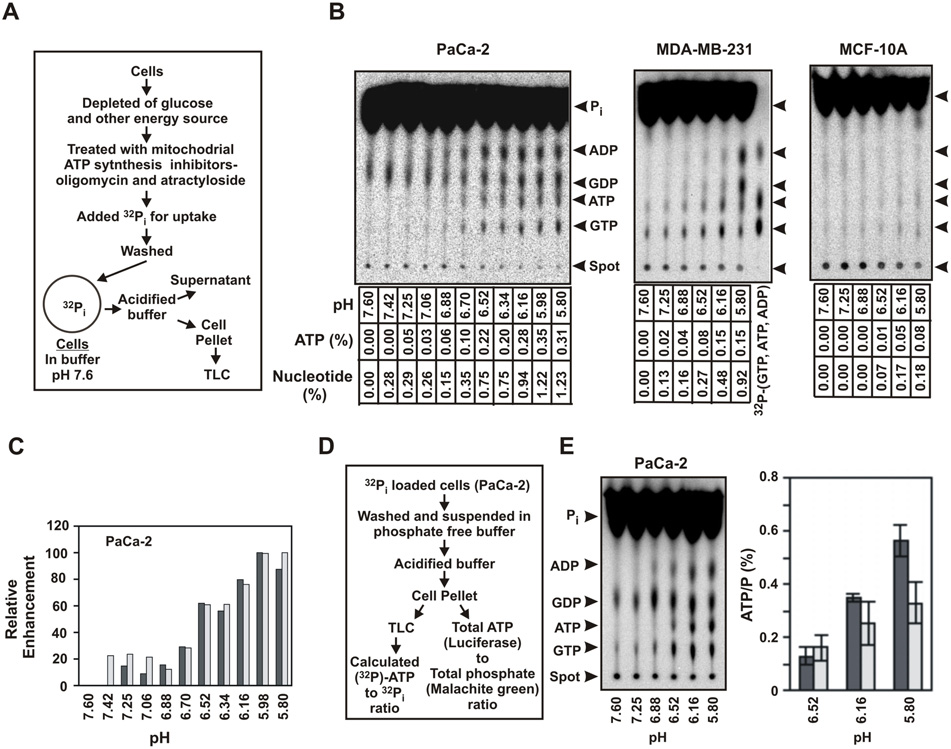
Synthesis of nucleotides from ^32^P_i_ in response to extracellular acid. (A) Schematic for preparation of ^32^P_i_ loaded cells for phosphate bond synthesis assay.(B) TLC analysis of the intracellular products of ^32^P_i_ loaded cells in response to acid gradient. Cells (1.75 million, 10 million/ml) at pH 7.6 were acidified to the indicated pH for 90 seconds. The cell pellets were separated, lysed with chloroform, extracted with buffer EB and used for TLC analysis. ATP and total nucleotide (ADP, ATP, GDP and GTP) formed are noted underneath the TLC as percent of total radioactivity, taking the values at pH 7.6 as zero. Effective elimination of ATP synthesis from glycolysis and mitochondria is indicated from the near-absence of any ATP at pH 7.6. Non-radioactive nucleotides were run as standard as separate spot or co-spotted on the samples in the same TLC plate. Available radioactive nucleotide standards were also used. The positions of the nucleotides are indicated by arrows. Production of radiolabeled nucleotides with extracellular acidification was tested at least three times. Confirmation of the nucleotides by characteristic enzymatic reactions is shown in S3 Fig. (C) Relative increase of ^32^P_i_ labeled ATP and total nucleotides with extracellular acid. Relative enhancement of ^32^P_i_ labeled ATP (black bar) and total nucleotides (grey bar) with pH are shown for PaCa-2 from the data in panel B. Maximum values of both ATP and total nucleotides were normalized to 100. (D) Schematic of the method to compare ATP synthesized from ^32^P_i_ and total increase in ATP. (E) Comparison of ATP/P_i_ obtained from TLC and from standard assay. Experiment was performed according to panel D. The cell pellets after chloroform lysis was extracted with 0.2 mM EDTA. TLC of PaCa-2 cell is shown. The ratio of ATP to P_i_ was measured from the TLC at pH 6.52, 6.16 and 5.8 that showed clear ATP bands (dark bars). The absolute amounts of ATP and phosphate in the same samples for each pH were also determined using luciferase assay and malachite green assay respectively. The ratio of increase in ATP to phosphate was determined (light bars). The bars represent average values of ATP/P (as percent) at the indicated pHs of two separate experiments (error bars = 2.s.d.).

In order to assess this, cell were first depleted of glucose and other metabolites by incubating at 37°C for 30 minutes in glucose-free buffer, and then treated with mitochondrial ATP synthesis inhibitors (5 μM oligomycin and 1 μM atractyloside). They were then allowed to uptake radioactive phosphate (^32^P_i_). The pH of the incubation buffer was 7.6 and had a total phosphate concentration of 1 mM. Depletion of glucose prevents formation of ATP and other glycolytic intermediates during incubation of ^32^P_i_. The ^32^P_i_ loaded cells were washed several times to remove free radioactivity and used for assay. After a brief acidification for 90 seconds, the cell pellets were separated from the supernatant, lysed and analyzed by PEI-cellulose thin layer chromatography (TLC). A brief schematic of the experiment is shown in Fig 3A. Apart from the expected band of ^32^P_i_, four major radioactive bands could be detected in the TLC in response to extracellular acidification. These bands were identified as ADP, ATP, GDP and GTP by their co-migration with standard references (Fig 3B) and also from the characteristics enzymatic reactions that corresponded to their identity (S3 Fig).

In the above experiments, effective prevention of ATP formation from either glycolysis or mitochondria was evident from the low intensity of radioactive ATP in the TLC at pH 7.6, even after prolonged incubation with ^32^P_i_ (Fig 3B). The band intensity of all the four nucleotides including ATP increased with decreasing extracellular pH for the aggressive cancer cell lines PaCa-2, MDA-MB-231 (Fig 3B) while a weak increase was observed in the immortalized cell line MCF-10A at the higher end of acidification (Fig 3B). No such products were seen in normal breast cell line HMEC. The formation of the nucleotides in response to acid gradient was strictly intracellular, as no product was detected in the supernatant. Retention of ^32^P_i_ by the cells suggests that cell membranes are not compromised under this condition. The total amount of radioactivity was used as a measure of intact cells and the amounts of products formed were normalized against these values. The amounts of labeled ATP and other nucleotides formed (ADP, GDP and GTP) were estimated as the percentage of total radioactivity and are noted underneath the TLCs (ATP and total nucleotide formation). The pH range over which the nucleotides were synthesized were similar to that seen in Fig 2B. The extracellular pH in malignant tumors can be as low as 6.1 [3]. Therefore, nucleotide synthesis below pH 6 would be indicative of cellular potential but may not be physiologically relevant to the cells, especially MCF-10A cell lines that do not thrive in acidic environment. The cell pellets from MCF-10A retained radioactivity under this condition, which indicated that the inactivity is not due to the compromise of the cell membrane under this condition. The formation of other nucleotides (apart from ATP) from free phosphate in response to extracellular acid suggested that the actual amounts of phosphate bonds formed was much higher than what we anticipated in the previous experiments in Fig 2 by just measuring ATP. The total phosphate bonds formed were almost four times that of ATP alone at this condition and showed a similar trend of increase with extracellular pH (Fig 3C). The formation of both nucleotide diphosphates (NDP) and triphosphates (NTP) were not surprising as the cells have ADKs and NDKs that would result in their inter-conversion. Their total increase in radioactivity is the measure for the net formation of new phosphate bonds.

Next, we wanted to see whether the entire enhancement in ATP upon acidification of the cancer cells resulted from the synthesis of new phosphate bonds (schematic, Fig 3D). PaCa-2 cells were loaded with ^32^P_i_ and washed as before. In addition, the cells were quickly washed once with phosphate-free buffer at pH 7.6, and then suspended in the same phosphate-free buffer for assay. Phosphate-free condition allowed for accurate determination of the amounts of total free phosphate contained in the cells, which can then be related to the radioactivity of the phosphate band in the TLC. The cells were acidified and the cell pellets were analyzed as before for nucleotides and P_i_ by TLC (radioactive bands). The ratio of radioactivity of ATP to P_i_ band in the TLC represents the ratio of the newly synthesized ATP (new phosphate bond) to that of the total free phosphate (Fig 3E). We also ascertained the ratio of the absolute values of enhancement of ATP and total free phosphate in the same sample using luciferase assay and malachite green assay respectively. Contribution of other processes to ATP synthesis would increase the latter ratio. As seen in Fig 3E, both the ratios were found comparable. This experiment confirmed that the increase in ATP levels upon acidification of cancer cells was almost entirely due to synthesis of new phosphate bonds from free phosphate.

### Acid gradient developed by gradual glucose metabolism can drive phosphate bond synthesis

**Fig 4:**
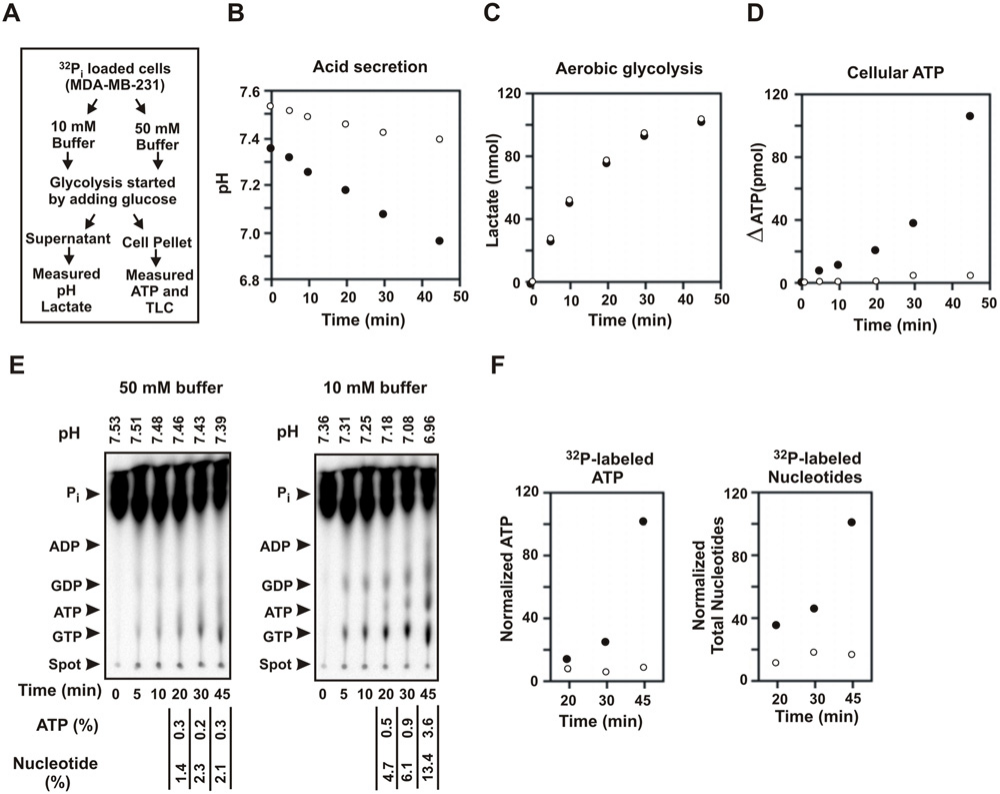
Acid gradient created by glucose metabolism promotesphosphate bond synthesis. (A) Schematic showing the steps followed to analyze the role of acid gradient in phosphate bond synthesis during glucose metabolism in cancer. MDA-MB-231 cell suspensions depleted of glucose and inhibited for mitochondrial ATP synthesis were loaded with ^32^P_i_, washed and suspended in high buffer capacity medium (50 mM) and low buffer capacity medium (10 mM) at pH 7.6. Glycolysis was initiated by adding 5 mM glucose. Aliquots were taken out at the indicated time and analyzed for extracellular pH, ATP, lactate production and radioactive nucleotide synthesis. Solid circle represents 10 mM buffer and open circle represents 50 mM buffer in all the panels. (B) Extracellular pH during glycolysis. The pH of the extracellular media was measured from the supernatant. (C) Rate of aerobic glycolysis. The amount of lactate formed was measured from the supernatant. (D) Increase in ATP levels in the cells. ATP levels were measured from the cell pellet using the aqueous extract after chloroform lysis. (E) Synthesis of nucleotides from ^32^P_i_ during aerobic glycolysis. The aqueous layer obtained from cells pellets in panel D were analyzed by TLC. Arrows show the positions of the nucleotides as determined by standard references. The extracellular pH measured for each time points (from panel B) are shown on the top. Amounts of ^32^P_i_ that converted to ATP and total nucleotides are shown below as ATP (%) and nucleotide (%). The values at start of glycolysis were taken as zero. The values for the time points 20 minutes and onwards were shown, as the bands can be reliably measured from that point. (F) Relative synthesis of ATP and total nucleotides. A comparison of the relative synthesis of ATP and total nucleotides are shown. The values (%) from panel E are normalized taking the highest value for 10 mM buffer at 45 minutes as 100. Experiments observing decrease in ATP synthesis in high buffer capacity medium as compared to low buffer capacity medium during acid secretion were repeated three times with similar results.

We then wanted to investigate whether natural acid gradient created by the slow and spontaneous secretion of acid, as observed in the physiological tumor microenvironment, could drive the synthesis of phosphate bonds from phosphate in cancer cells. We used ^32^P_i_ loaded cells to monitor phosphate bond synthesis, and glucose metabolism to create the natural acid gradient. The experiment was performed according to the schematic shown in Fig 4A. Briefly, MDA-MB-231 cells that were depleted of glucose and containing mitochondrial ATP synthesis inhibitors (5 μM oligomycin and 1 μM atractyloside) were loaded with ^32^P_i_ and washed as described previously. They were then separately suspended in high buffer capacity medium (containing 50 mM Hepes-MES) and in low buffer capacity medium (containing 10 mM Hepes-MES) at pH 7.6. Each buffer also contained the mitochondrial inhibitors and 1 mM sodium phosphate. During acid secretion by the cells, the medium with high buffer capacity will allow lesser changes in extracellular pH while the medium with low buffer capacity will allow greater changes in extracellular pH. Glycolysis was started by adding 5 mM glucose. Aliquots were taken out at desired time intervals and quickly centrifuged to separate the cell pellet and the supernatant. The supernatant was used to measure the pH of the media and the amount of lactate formed. The cell pellets were immediately lysed and was used for the estimation of ATP by luciferase assay and for TLC analysis. The results of this experiment are shown in Fig 4B–4F). The high buffer capacity media exhibited significantly less decrease in pH (7.53 to 7.39) compared to the low buffer capacity media (7.36 to 6.96) (Fig 4B). The rate of aerobic glycolysis as measured from lactate formation was found to be identical in both cases (Fig 4C). However, the ATP levels in the cells increased at a much faster rate with time in the low buffer capacity media (10 mM) where there was a greater decrease in extracellular pH as compared to the high buffer capacity media (50 mM) (Fig 4D). TLC analysis of the aqueous extract of the cell pellet revealed that much higher amounts of radioactive ATP and other nucleotides were synthesized from ^32^P_i_ in the 10 mM media compared to the 50 mM media (Fig 4E). A prominent band of ATP appeared in 10 mM media at around 20 minutes at an extracellular pH of 7.2 and increased in intensity with decreasing pH of the medium. A comparison of the relative amounts of ATP and total nucleotides formed at each of the time points in the 50 mM and 10 mM buffers are shown which clearly shows a sharp increase in radio-labeled ATP and total nucleotides with decrease in extracellular pH (Fig 4F).

This experiment indicated that the acid gradient developed by gradual glucose metabolism, as in seen in tumor, can drive synthesis of phosphate bonds.

### Phosphate bond synthesis activity is present in the plasma membrane of cancer cells

**Fig 5:**
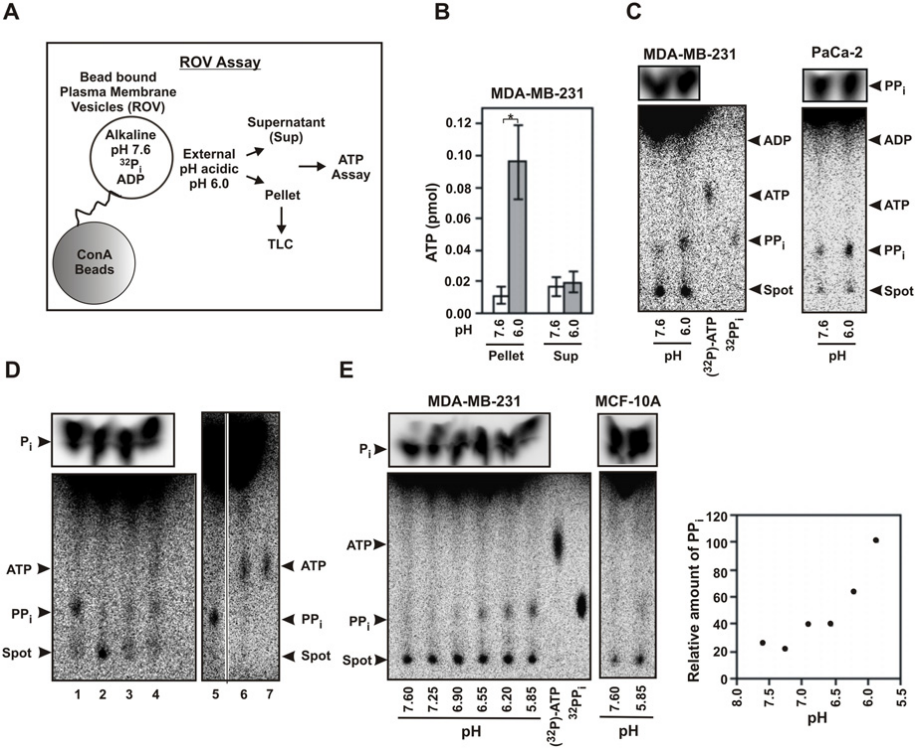
Plasma membrane vesicles (ROV) of cancer cells can produce ATP and PP_i_ with acid gradient. (A) Schematic of the experiment showing assay of bead-bound ROV. The internal pH of the vesicles were kept alkaline (pH 7.6) while the outside was made acidic. (B) Plasma membrane vesicles of cancer cells can produce ATP with acid gradient. Bead-bound ROV from MDA-MB-231 loaded with P_i_ and ADP at pH 7.6 were washed and acidified to pH 6.0 for 90 seconds. The supernatant was removed and the vesicles bound to the beads were lysed, extracted with buffer EB and the amounts of ATP formed were estimated both from the pellet and the supernatant (sup) by luciferase assay. The bars represent the mean from 7 experiments (error bars = 2.s.d. The p values were calculated using student’s two-tailed test: *p<0.05). (C) Plasma membrane vesicles of cancer cells can also produce PP_i_ from P_i_. TLC of the ROV experiment is shown. The load controls are shown above at a lower exposure. The productions of PP_i_ with transmembrane acid gradient of ROV as shown above were done ten times showing similar results. (D) Confirmation of PP_i_ band by enzyme reactions. Aqueous fraction from PaCa-2 plasma membrane vesicles at pH 6.0 similar to Panel C were analyzed by enzyme reactions for the confirmation and identification of PP_i_. The aqueous fraction was diluted with 4 volumes of buffer (10 mM tris pH 8.0, 0.2 mM EDTA and 1 mM MgCl_2_), distributed into equal aliquots, and were subjected to PP_i_ specific enzymatic reactions and analyzed on TLC. Lane 1, untreated sample; lane 2, phosphatase (P-ase): PP_i_ band decreased; lane 3, pyrophosphatase (PP_i_-ase): PP_i_ band decreased; lane 4, nicotinamide mononucleotide adenylyl transferase 1 (NMNAT1) and excess NAD: PP_i_ band decreased and ATP band appeared; lane 5, untreated sample; lane 6, ATP sulphurylase and excess Adenosine-5’- phosphosulphate (APS): PP_i_ band decreased and ATP band appeared. Lane 7, (γ-^32^P)-ATP as standard. (E) Plasma membrane vesicles of cancer cells can produce PP_i_ from P_i_ in response to acid gradient. Experiments similar to panel C were done with ^32^P_i_ only and in absence of ADP. Intensity of PP_i_ bands were corrected for load by intensity of ^32^P_i_ and then represented as relative increase with the maximum as 100. The pH profile was repeated three times. Two point test (single addition of acid to pH 6.0) yielding PP_i_ was performed more than ten times showing similar results. Time kinetics of PP_i_ formation from P_i_ is shown in S5 Fig.

In order to further confirm that the phosphate bond synthesis activity in response to acid gradient is present in the plasma membrane of cancer cells, we performed these experiments in cytosol-free purified plasma membrane vesicles (pm-vesicles). Vesicles were prepared from purified plasma membrane preparations that were free of cytosolic or mitochondrial membrane contamination, as determined from immunoblot. They were sealed in appropriate buffer of pH 7.6 containing radioactive phosphate (^32^P_i_) and ADP (see methods). Membranes can seal as rightside-out vesicle (ROV) or inside-out vesicle (IOV). The ROV were isolated from IOV by binding the former to concanavalin A (conA) beads as reported by other investigators [31]. The sealed vesicles bound to the beads were washed several times, acidified for 90 seconds at indicated pH, centrifuged and the supernatant and pellet were separated. The vesicles bound to the beads were then lysed and assayed for ATP with luciferase assay. The schematic of the experiment is shown in Fig 5A. The pm-vesicles from cancer cells were found capable of synthesizing ATP as shown for MDA-MB-231 in Fig 5B. No ATP was found in the supernatant that was recovered after separation of the beads. This indicated that the synthesis of ATP occurred inside the vesicles. Production of ATP was undetectable with MCF-10A vesicles under similar conditions. The small amounts of ATP formed were difficult to detect reliably in PEI cellulose TLC as shown in Fig 5C for MDA-MB-231 and PaCa-2. However, the TLC revealed a very prominent radioactive band in response to acid that migrated with pyrophosphate (PP_i_). The identity of the band as PP_i_ was confirmed using enzymes that were very specific to PP_i_ (Fig 5D) [32]. Therefore, it appeared that even though ATP was produced by acid gradient driven condensation of ADP and P_i_ as seen from luciferase assay (Fig 5B), there was also a possibility of phosphate bond formation by the direct condensation of two P_i_ moieties to form PP_i_. Alternatively, one could postulate that the PP_i_ was formed from the hydrolysis of synthesized ATP at the α-β phosphate linkage, which was next assessed.

In order to confirm that PP_i_ was produced by the direct condensation of P_i_, pm-vesicles were prepared at pH 7.6 as the previous experiment in the presence of ^32^P_i_ only, but without any ADP. It was found that pm-vesicles from cancer cells were indeed capable of producing PP_i_ from P_i_ in response to acid gradient. PP_i_ formation in the vesicles started at around an external pH of 6.9 and reached maxima at around pH 6 as seen in Fig 5E for MDA-MB-231. The pm-vesicles from non-cancer cell line MCF-10A gave a very feeble response (Fig 5E). Time kinetics at an internal pH of 7.6 and an external pH of 6.4 revealed a prominent band of PP_i_ within 30 seconds of reaction that increased gradually till about 2 minutes (S4 Fig).

The above experiments confirmed that the purified plasma membrane vesicles from cancer cells have the ability to form ATP by condensation of ADP and P_i_ and also to form PP_i_ by condensation of two P_i_ in response to acid gradient. The latter reaction appears to be more dominant, at least in the context of purified membrane and in the absence of cytosol.

### IOV assay with reverse acid gradient and role of cytosol in ATP synthesis

**Fig 6:**
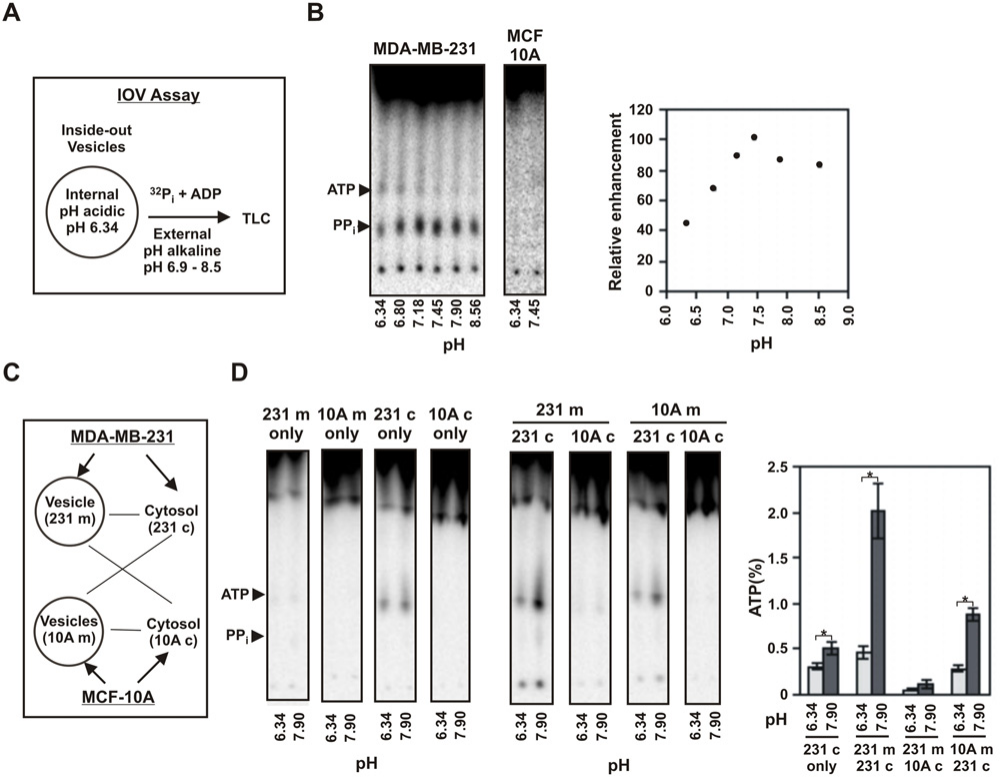
Plasma membrane vesicles (IOV) and cytosols from cancer cells together can form ATP. (A) Schematic showing the procedure for IOV assay with reverse acid gradient. Vesicles were sealed at pH 6.34. To this was added ^32^P_i_ (300 μCi/ml, 0.1 mM) and ADP (0.1 mM) and the pH was shifted to the indicated pH by adding small aliquot of alkali. The reaction was quenched after 90 seconds and then analyzed by TLC. (B) Formation of PP_i_ by inside-out vesicles (IOV) with reverse acid gradient. Intensity of PP_i_ bands were corrected for load by intensity of ^32^P_i_ and then represented as relative increase with the maximum as 100. The pH profile was repeated three times. Two point test (single addition of alkali to pH 8) yielding PP_i_ was performed more than ten times with similar results. (C) Schematic showing IOV and cytosol combinations used in panel D and the abbreviations used. Vesicles were sealed at pH 6.34. To this was added ^32^P_i_ (200 μCi/ml, 0.2 mM) and ADP (100 μM). Cytosolic extract (5 μg protein) at pH 7.5 was added to 25 μl reaction and the pH was immediately shifted to the indicated pH by adding small aliquot of alkali. The reaction was quenched after 10 seconds and analyzed by TLC. (D) IOV and cytosol combination experiment. The TLCs of the experiment are shown. The amount of ATP (%) formed relative to ^32^P_i_ were estimated and are represented as bars. The data represents mean of 3 similar experiments (error bar = 2.s.d. The p values were calculated using student’s two-tailed test: *p<0.05).

In our radioactive experiments with viable cells in Figs 2 and 3, ATP were formed while in the vesicles we observed predominant formation of PP_i_ and much less formation of ATP. This indicated probable involvement of cytosolic factors in the formation of ATP. The rightside-out vesicle (ROV) assay shown in Fig 5 has the advantage of working with purified sealed vesicles and to demonstrate that ATP and PP_i_ synthesis are occurring inside the vesicles. However, it has the limitation that all reactants have to be packed into the vesicles before binding to the beads. We therefore designed an assay for the inside-out vesicles (IOV) that would allow us to add the cytosol to the sealed vesicles for a short period of time under a reverse acid gradient. We first tested the efficacy of the IOV assay in forming phosphate bonds with reverse acid gradient. Vesicles prepared from the plasma membrane constitute a mixture of IOV and ROV. We prepared sealed membrane vesicles at an acidic pH of 6.34. ADP and ^32^P_i_ were added to it and the pH was immediately made alkaline (pH 6.8–8.5) by adding alkali. After incubation for 90 seconds, the vesicles were lysed with chloroform and checked for ATP and PP_i_ formation by TLC (schematic, Fig 6A). We observed formation of PP_i_ with reversed acid gradient that peaked at an external pH of around 7.5–7.9 when the internal pH was 6.34. As before, no prominent band of ATP was detected (Fig 6B).

In order to assess the role of cytosol in facilitating synthesis of ATP, we used plasma membrane vesicles and cytosols from both MDA-MB-231 (cancer) and MCF-10A (normal, immortalized). The ability of the vesicles from each of these cell lines to produce ATP with acid gradient was tested in the presence of the cytosols obtained from each of the cell lines (Fig 6C and 6D). As expected, we observed that the membrane vesicles alone from both the cell lines showed no formation of ATP with change in pH. The cytosol from MDA-MB-231 showed some initial ATP but did not show any significant enhancement with change of pH while the cytosol from MCF-10A showed no ATP formation. However, in the combination experiment, the vesicles from MDA-MB-231 gave significant amounts of ATP with the cytosol from MDA-MB-231 and almost none with the cytosol from MCF-10A when the pH was made alkaline. The vesicles from MCF-10A gave no response with cytosol from MCF-10A but gave a descent response with the cytosol from MDA-MB-231, albeit much weaker compared to the vesicle from MDA-MB-231. It may be noted that the cytosol of MDA-MB-231 has a background formation of ATP. This is because the vesicles do not get completely removed upon ultracentrifugation during cytosol preparation. The contaminating vesicles have an alkaline internal pH of 7.4, and therefore form ATP when added to the reaction mixture at pH 6.34. However, they do not respond to further alkalization of the reaction mixture.

This experiment shows that the plasma membrane and cytosol from cancer cells in combination have enhanced ability to synthesize ATP in response to acid gradient. ATP may be formed directly by condensation of ADP with P_i_ as was seen earlier with vesicle alone (Fig 5B) which may get accelerated or favored in presence of cytosol. In addition, PP_i_ being the predominant product in the vesicle assay, one can also contemplate a parallel reaction of transient intermediate formation of PP_i_ from P_i_ by acid gradient that is subsequently being converted to ATP by the cancer cytosol. This would however require that the cancer cytosol would be able to convert PP_i_ to ATP which was next tested.

### Cytosols of cancer cells can convert PP_i_ to ATP

**Fig 7:**
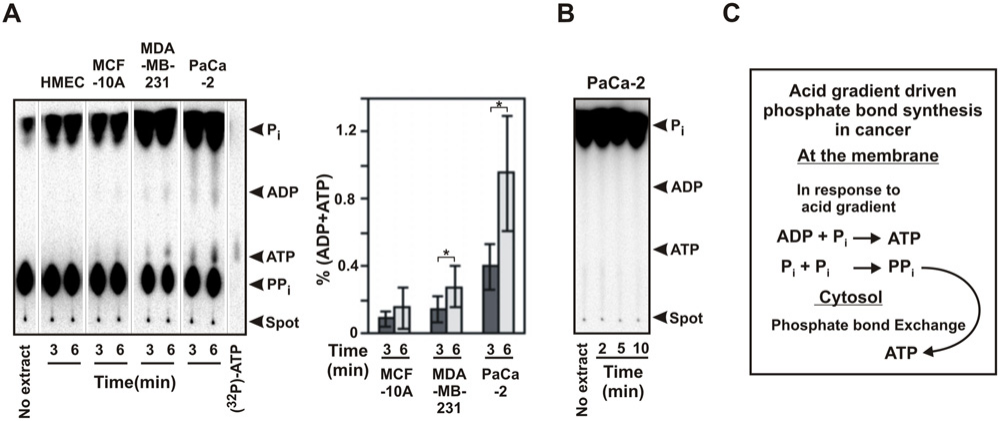
Cytosols of cancer cells can convert PP_i_ to ATP. (A) Synthesis of ATP from ADP and ^32^PP_i_ by cancer cytosols. Cytosolic extracts (2 μg protein) at pH 7.5 were added to 50 μl reaction mixture containing 10 mM Tris pH 7.5, 1 mM NaH_2_PO_4_, 100 mM NaCl, ^32^PP_i_ (200 μM, 30 μCi/ml), 1 mM MgCl_2_ and 0.25 mM ADP at pH 7.5. The reactions were quenched after indicated time and analyzed on TLC. Some ADP was also formed. The bar graph represents the percent of PP_i_ (total radioactivity) that converted to ATP and ADP (error bars = 2.s.d., n = 3–4. The p values were calculated using student’s two-tailed test: *p<0.05). (B) No products were obtained from ADP and ^32^P_i_ by cancer cytosols. Reaction are performed with cytosol from PaCa-2 similar to panel A (performed three times) but using ^32^P_i_ (200 μM, 1 mM) and 200 μM PP_i_ (no ^32^PP_i_). (C) Schematic showing the plausible steps in the formation of ATP by acid gradient indicating the role of membrane and cytosol in the process.

Cytosols from the cell lines (HMEC, MCF10A, MDA-MB-231, PaCa-2) were prepared as described in the methods. They were then tested for their ability to convert radioactive ^32^PP_i_ and ADP into ATP (Fig 7A). We observed that the cytosol from cancer cells MDA-MB-231 and PaCa-2 could produce radio-labeled ATP from ^32^PP_i_ while cytosol from normal immortalized cells MCF-10A did so to a much lower extent. The cytosol from normal breast epithelial cells HMEC failed to generate any ATP. Some radio-labeled ADP was also detected. The relative rates for conversion of PP_i_ was found to be about two-fold higher in MDA-MB-231 and six-fold higher in PaCa-2 as compared to that for MCF-10A. Radioactive ^32^P_i_ failed to give any ATP under similar condition, which confirmed that ATP was indeed produced from PP_i_ (Fig 7B). Therefore, it appears that unlike cytosols of normal cells, the cytosols of cancer cells have the ability to convert PP_i_ to ATP.

Cytosols from cancer cells had a very high PP_i_-hydrolysis activity as compared to cytosols from normal cells or immortalized cells, manifestation of which could be seen from the production of free ^32^P_i_ from ^32^PP_i_ during the reactions (Fig 7A). Under similar condition (γ-^32^P)-ATP remained unaltered (data not shown). This indicated that the hydrolysis of PP_i_ was due to specific PP_i_-ase activity and not from phosphatase activity. It appeared that if not converted to nucleotides, the free PP_i_ in the cell would be rapidly hydrolysed. This also re-affirms that the higher amount ATP seen in cancer cells upon extracellular acidification as compared to normal cells is not due to less phosphate-bond hydrolysis activity of the cancer cytosols but rather due to greater synthesis of phosphate bonds in cancer cells.

## Discussion

Proton-motive force is one of the most widely used energy-source for all biological systems to generate cellular chemical energy [15, 17]. Physiological evidence associating acid gradient to proliferation of cancer cells has been demonstrated by other investigators both in-vitro and in-vivo, which also indicated that under such condition cancer cells can support its energy demand [8, 9, 21]. In this work, we have presented biochemical evidence that cancer cells have the potential to utilize extracellular acid gradient as an energy source to synthesize high-energy phosphate bonds. This function of acid gradient is in addition to other growth advantages conferred to cancer cells by the extracellular acidic pH through gene regulation, metabolic reprogramming and regulation of cell cycle; and may partly explain how these highly proliferative cancer cells support their energy needs. Use of cultured cell lines, purified plasma membrane and radioactivity allowed us to confirm phosphate bond synthesis independent of the contribution from glycolysis and mitochondria, which would not be possible in an in-vivo system.

The mechanism of intracellular production of ATP in response to inwardly- directed acid gradient reported here is different from the one reported for ectopic ATP synthesis by plasma membrane ATP synthase that requires addition of ADP into the external media [33, 34]. In the latter case, the synthesis of ATP is extracellular and is believed to be so since the catalytic moiety is oriented outward. Increase in activity with lowering of pH of the medium was reported for this system in cancer cells [34] but how an inwardly-directed acid gradient drives synthesis of ATP on the outside (energetically unfavorable) is not very clear. A possible involvement of ecto adenylate kinase rather than ecto ATP synthase in the process is speculated [35]. In our experiments, we demonstrated intracellular production of radioactively labeled nucleotides and pyrophosphates from radioactive phosphate in response to inwardly-directed acid gradient, as would be expected from the stand point of energetics. Formation of radioactively labeled nucleotides and pyrophosphate from radioactive phosphate is a confirmative demonstration of the synthesis of phosphate bonds in response to acid gradient.

### Potential to trap the energy of acid gradient is located in the plasma membrane of cancer cells

Experiments with vesicles and radioactive phosphate confirmed that the ability to trap the energy of acid gradient as phosphate bond is located in the plasma membrane of cancer cells and is absent in normal cells. Phosphate bonds can be formed by the condensation of ADP and P_i_ to form ATP or by the condensation of two P_i_ to form PP_i_ (Figs 5 and 6). The cytosols of cancer cells have the ability to enhance the formation of phosphate bond in the form ATP either by facilitating the former reaction or by converting PP_i_ to ATP by phosphate bond exchange (Figs 6 and 7). A schematic depiction of the plausible steps is shown in Fig 7C. ATP is subsequently converted to other nucleotides in the cytosol by the action of ADKs and NDKs.

### Transient synthesis PP_i_ as an intermediate may be energetically favorable

The mechanism by which the energy of the acid gradient is translated into chemical energy of phosphate bonds or the nature of the enzyme(s) that catalyze the process is not clear. Whether the enzyme(s) act in a similar fashion as the ATP-synthases giving both PP_i_ and ATP remains to be investigated [36]. However, failure of oligomycin (bonafide ATP synthase inhibitor) to inhibit the reaction suggests fundamental differences. The energy required for the synthesis of PP_i_ from P_i_ is lower than that required for the synthesis of ATP from ADP. The ΔG°PP_i_ is 21.6KJ/mol while the ΔGATP is 50.9 KJ/mol at an ATP/ADP ratio of 7.42 that is normally found in the cell [37]. Both the high concentration of P_i_ in the cell (10–12 mM) and the stoichiometry (two P_i_ per PP_i_) would make the synthesis of PP_i_ more favorable. Therefore, from thermodynamic consideration, transient formation of PP_i_ from P_i_ may be a more favorable reaction compared to the direct synthesis of ATP from ADP and P_i_. In presence of cancer cytosol, this thermodynamic balance is either altered in favor of direct synthesis of ATP, or the PP_i_ that is transiently formed gets converted to ATP by phosphate transfer reaction (Figs 6 and 7). In fact, direct synthesis of PP_i_ from P_i_ had been demonstrated in the chloroplast of plants and phototropic bacteria [13, 38]. This PP_i_-synthase is a single subunit protein of 660 amino acids with 15 transmembrane segments [38]. This suggests that acid gradient driven phosphate bond synthesis can also be carried out by structural motifs other than the rotary pump structure seen in F_1_-F_o_ ATP-synthase [36].

### Properties of the phosphate bond synthesis and its significance to acidic tumor milieu

The optimum range of extracellular pH for phosphate bond synthesis was found to be from 6.0 to 7.2 (Figs 2 and 3). This is most suited in the context of cancer microenvironment whose pH also lies in a similar range, 6.15 to 7.4 [3]. The rate of acid driven ATP synthesis at pH 6.7 was determined to be about 5.5 nmol/min/million-cells. The total phosphate bond synthesis (considering all nucleotides) can be even higher (Fig 3). The rate of ATP flux from glycolysis at pH 7.5 was measured to be 4.95 nmol/min/million-cells (Fig 1). At pH 6.7, the rate from glycolysis actually decreased [glucose consumption rate: 1.93 nmol/min/million-cells (s.d. = ±0.19, 6 data sets); lactate production rate: 2.88 nmol/min/million-cells (s.d. = ±0.2, 6 data sets)]. Both the rates were measured in presence of oligomycin. Comparison of the rates indicate that the acid gradient can be a major source of energy in cancer; especially for those cancer types that have switched predominantly towards glucose to lactate fermentation and have an acute acidic environment. Extracellular acid may be contributed by tumor cells different from the ones that are utilizing it for phosphate bond synthesis. Natural acid gradient created through slow and spontaneous acid secretion during aerobic glycolysis, as seen in physiological situation in tumor, can drive phosphate bond synthesis in cancer cells (Figs 1 and 4). This strongly suggests that the natural acid gradient found in tumor milieu has the potential to play a similar role in phosphate bond synthesis.

### An integrated view of aerobic glycolysis and extracellular acid gradient in cancer

Aerobic glycolysis confers many strategic advantages to cancer cells. However, applying our traditional view of glycolysis that gives two molecules of ATP per glucose, aerobic glycolysis would be considered a very inefficient method for generation of energy. The levels of ATP in cancer cells are believed to be maintained by enhanced uptake of glucose through the up-regulation of glucose transporters [39]. Increased glucose consumption would result in increased acid secretion, further reducing the pH of the extracellular milieu. Increased proton-motive force created across the plasma membrane due to the increased extracellular acidification would drive higher rate of synthesis of phosphate bonds by the plasma membrane phosphate-bond synthesis machinery. Therefore, aerobic glycolysis as an energy source in cancer can be considered as an integrated mechanism consisting of the fermentation of glucose to lactate, secretion of acid and then utilization of the acid gradient to produce high-energy phosphate bonds. This makes aerobic glycolysis relatively more energy efficient than is popularly believed. Conversion of glucose to lactate is a faster method for generation of energy as compared to multiple step mitochondrial ATP production and is often the method of choice during intense need [12]. In addition, aerobic glycolysis accompanied by acidification of external milieu confers strategic advantage to cancer cells in term of metabolite reprogramming [5–7], increased invasiveness [8–10] and regulation of cell cycle [11]. Our works identifies another additional advantage to the process in terms of energy generation.

In mitochondria and chloroplast, the acid is retained in the inter membrane space due to their double membrane structure [12]. On the other hand, in tumors, ineffective perfusion causes accumulation of the secreted acid. More aggressive and metastatic cells are comparatively more exposed to blood and can be expected to have better perfusion [3, 40]. During our experiments, we have observed that if the cancer cell suspensions were not stirred during acid secretion upon glucose consumption, the ATP levels increased very rapidly due to local acidification at the vicinity of the cancer cells. However, moderate stirring that keep the cells in suspension decreased the spike in ATP levels but could not eliminate it. This is more prominent with PaCa-2 cell lines which are very sensitive to acid gradient due to their second order kinetics. Rapid stirring (or diffusion rate) is necessary for immediate homogenous distribution of the secreted acid in order to achieve a uniform increase in ATP with time with decrease of pH of the bulk media. Therefore, due to the high efficiency and sensitivity of the plasma membrane phosphate-bond synthesis machinery in cancer cells, one can speculate that the metastatic cells in contact with blood will produce ATP depending on how fast and efficiently the acid gradient gets dissipated from the vicinity of the cell membrane.

The dynamics of hydrogen ions (protons) in facilitating the synthesis of the phosphate bonds remains to be investigated. If actual flow of proton is necessary to drive the phosphate bond synthesis as seen for ATP synthase [36], then it would also be necessary to extrude the proton by passive processes that would not require hydrolysis of phosphate bonds, so as to preserve the overall benefit of the process. Such passive extrusion of proton can be facilitated by the plasma membrane associated monocarboxylate transporters and carbonic anhydrases, whose enhanced activity and importance in cancer has already been established [41]. Moreover, involvement of other hitherto unidentified proton extrusion machinery cannot be ruled out.

### General importance and implications

Our work demonstrates the ability of aggressive cancer cells to utilize plasma membrane proton-motive force to synthesize phosphate-bonds. Apart from its importance to cancer, the fact that such novel activity is at all present in a mammalian cell has its own significance. The mechanism by which this activity gets associated with the plasma membrane with gradual neoplastic transformation of cells is an important and interesting question to pursue. A weak and rudimentary activity was observed in the highly proliferative immortalized cell line MCF-10A. Therefore, other highly proliferating cells being able to generate cellular energy through similar mechanisms, albeit to a limited extent, and under limited conditions, remains a possibility. One can speculate different reasons for the appearance of this activity. This activity may be due to genetic alteration in cancer. It may also be due to post-translational or chemical modification of existing enzymes in the oxidative environment of aggressive cancer cells. The robust activity seen in our assay suggests that the plasma membrane phosphate bond synthesizing machinery and its associative cytosolic counterparts could be potential therapeutic targets against aggressive cancers.

## Supporting Information

S1 Fig Mathematical model for steady state ATP synthesis in response to extracellular acid. (PDF)

S2 Fig Determination of order of reaction (n). Plot of log (A/A_o_-1) against pH for MDA-MB-231, A549 and PaCa-2 are shown for the dataset from Fig 2B. A and A_o_ are the ATP levels at the indicated pH and at pH 7.5 respectively. Data points in the exponential portion of the curve were taken (see supplementary information for details). The average values of n (slope) from multiple experiments are: MDA-MB 231, 0.89 (s.d. = ±0.07, 7 data sets); A549, 0.79 (s.d. = ±0.06, 4 data sets) and PaCa-2, 2.12 (s.d. = ±0.23, 6 data sets).
(TIF)

S3 Fig Confirmation of the nucleotides bands by characteristic enzymatic reactions. The products of acidification of ^32^P_i_ loaded PaCa-2 cells similar to Fig 3B were diluted with 4 volumes of buffer (10 mM tris pH 8.0, 0.2 mM EDTA and 1 mM MgCl_2_) and subjected to different enzymatic reactions that are characteristic of the nucleotides as discussed in the methods. This is then subjected to TLC analysis. The enzymes and the substrates used for each characteristic reaction and their effects are noted for each lane (spot). The appearance (or increased intensity) or disappearance (or reduced intensity) of the bands upon specific reactions confirmed their identity. Lanes 1, untreated; lane 2, phosphatase (P-ase): all the nucleotides disappeared; lane 3, pyrophosphatase (PP_i_-ase): no change was observed. Lane 4, phosphodiesterase (PDE): cleaves at the α-β phosphate bond—generated PP_i_ from ATP and GTP which migrates at the same place as GTP and generated P_i_ from ADP and GDP; lane 5, treatment with PDE followed by PP_i_-ase: PP_i_ produced from PDE gets hydrolyzed by PP_i_-ase and the PP_i_ band disappeared—confirms that the product of PDE is PP_i_; lane 6, adenylate kinase (ADK) and excess AMP (1 mM): ATP diminished while ADP intensified; GTP, a weak substrate for ADK, also diminished; lane 7, nucleotide diphosphokinase (NDK) and excess ATP (1 mM): ADP and GDP diminished while ATP and GTP intensified; lane 8, NDK and excess ADP: GTP diminished; lane 9, ^32^P-(ADP, ATP, GTP) standard. ^32^PP_i_ migrates at the same position as GTP.
(TIF)

S4 Fig Time kinetics of PP_i_ formation from P_i_ in plasma membrane vesicles. Suspensions of plasma membrane vesicles from MDA-MB-231 loaded with ^32^P_i_ at pH 7.6 and bound to conA beads were acidified to pH 6.4 for the indicated amount of time, processed and analyzed by TLC as shown in the schematic. Time interval between addition of acid and lysis by chloroform was taken as the time of acidification. The load controls for ^32^P_i_ in the TLC are shown above at a lower exposure. The amounts of PP_i_ were corrected for the load using the P_i_ band intensity. Plot of relative amount of PP_i_ is shown taking the highest value as 100. Full range time kinetics was done 2 times but kinetics with less number of time points was done 4 times.
(TIF)
