# Supplementary material for: Acid Gradient across Plasma Membrane Can Drive Phosphate Bond Synthesis in Cancer Cells: Acidic Tumor Milieu as a Potential Energy Source
Source: PLoS One. 2015 Apr 15;10(4):e0124070. doi: 10.1371/journal.pone.0124070 (PMC4398327; doi:10.1371/journal.pone.0124070)
Supplement: S1 Fig — (PDF) [file pone.0124070.s001.pdf]

Mathematical model for steady state ATP synthesis in response to extracellular acid

To build the mathematical model, the following assumptions were made:

1. The rate of formation of ATP $(d[A]/dt)_H$ from plasma membrane in response to acid gradient is proportional to the nth order of the concentration of acid and is represented as $(d[A]/dt)_H = K_1.[H]^n$. Here [A] is the molar concentration of ATP, t is time, K_1 is the rate constant for the formation of ATP in response to acid and [H] is the molar concentration of acid. For simplicity we have considered that $[H] = [H]_{out} >> [H]_{in}$ and concentration of ADP is constant.
2. The rate of consumption of ATP, $(d[A]/dt)_c$, by all cellular enzymes (for useful work, intrinsic hydrolysis, phosphate bond transfer or all other means of consumption) is proportional to the concentration of ATP at that time and is represented as $(d[A]/dt)_c = K_2.[A]$. Here [A] is the molar concentration of ATP at that time. K_2 is the rate constant for the consumption of ATP.
3. The cell has an intrinsic rate (in absence of acid) of synthesis of ATP (r) which remained unchanged during the experiment.
4. When steady state is reached, the net rate of formation of ATP is equal to the net rate of consumption of ATP i.e., $(d[A]/dt) = 0$.

When there is no acid response, i.e. at around pH 7.5, cells have one source of ATP production which is the internal production (r). If the concentration of ATP at this

pH be $[A_o]$ then the rate of consumption of ATP is equal to $K_2.[A_o]$. The steady state at this pH can be represented by eq (1).

$r = K_2.[A_o]$ ......eq(1)

During acid response we have an additional source of ATP from the plasma membrane. The overall steady state during acid response can be represented as

$r + (d[A]/dt)_H = K_2.[A]$ ....eq(2) and from assumption 1 we can write,

$r + K_1.[H]^n = K_2.[A]$ ....eq(3)

Combining eq(1) and (3) and rearranging we can get to the form

$[A]/[A_o] - 1 = [H]^n.(K_1/K_2.[A_o])$ ......eq(4)

If A and A_o be the absolute amount of ATP for a given number of cells for which the cytosolic volume is V_c, then $[A] = A/ V_c$ and $[A_o] = A_o/ V_c$.

Therefore eq(4) can be written as

$A/A_o - 1 = [H]^n.(K_1.V_c /K_2.A_o)$ ......eq(5)

Taking log on both side and substituting pH = -log[H] we get,

$\log(A/A_o - 1) = -n.pH + \log(K_1.V_c /K_2.A_o)$ ......eq(6) and

$\log(A - A_o) = -n.pH + \log(K_1.V_c /K_2)$ ......eq(7)

Plot of $\log(A/A_o - 1)$ or $\log(A - A_o)$ against pH will give a straight line with a negative slope, the value of which will give the order of the reaction (n).

Eq(7) can also be written in the exponential form as

$A - A_o = (K_1.V_c/K_2).10^{-.n.pH}$ ......eq(7)

This represents how ΔA, the steady state enhancement of ATP for a given number of cells, varies with pH, which as can be seen, is an exponential relationship. Therefore the relationships and equations discussed above are valid in the exponentially rising

portion of the curve. It can also be applied to other nucleotides and entities that show similar dependence with pH.
