# Supplementary figures and images for: Acid Gradient across Plasma Membrane Can Drive Phosphate Bond Synthesis in Cancer Cells: Acidic Tumor Milieu as a Potential Energy Source
Source: PLoS One. 2015 Apr 15;10(4):e0124070. doi: 10.1371/journal.pone.0124070 (PMC4398327; doi:10.1371/journal.pone.0124070)

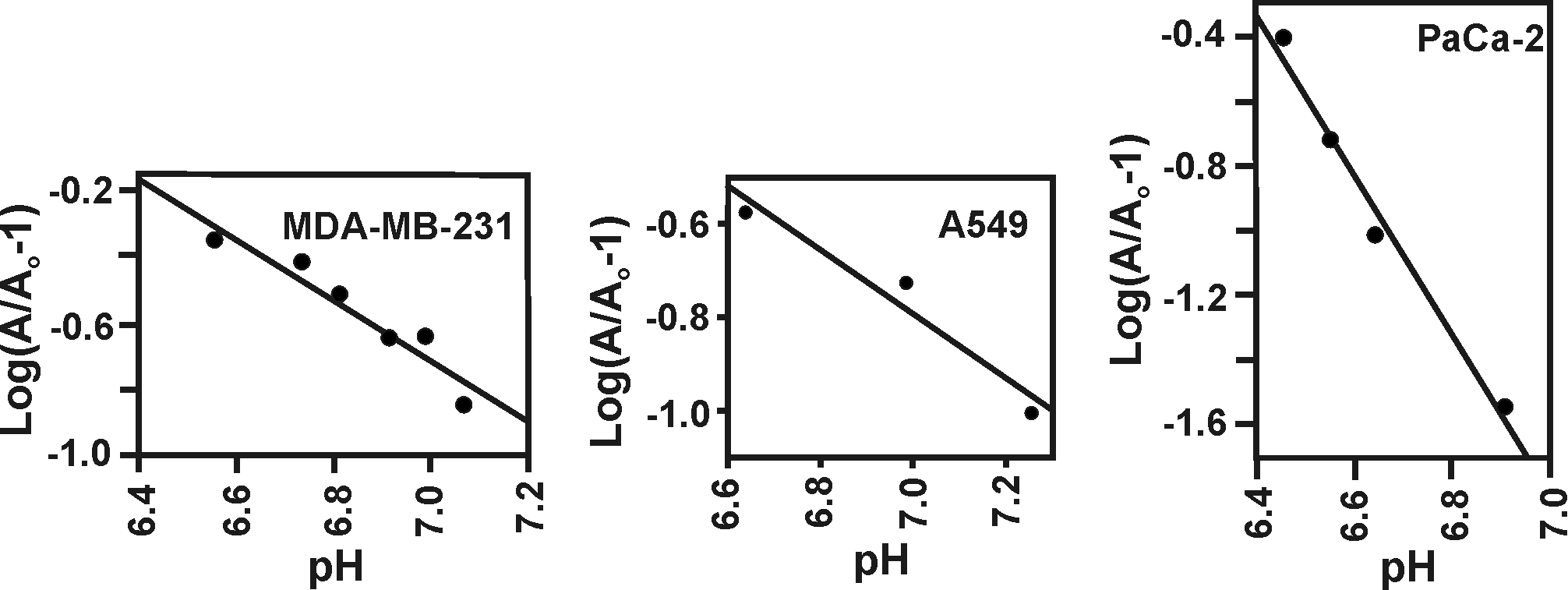

Supplement: S2 Fig — Plot of log (A/Ao-1) against pH for MDA-MB-231, A549 and PaCa-2 are shown for the dataset from Fig 2B. A and Ao are the ATP levels at the indicated pH and at pH 7.5 respectively. Data points in the exponential portion of the curve were taken (see supplementary information for details). The average values of n (slope) from multiple experiments are: MDA-MB 231, 0.89 (s.d. = ±0.07, 7 data sets); A549, 0.79 (s.d. = ±0.06, 4 data sets) and PaCa-2, 2.12 (s.d. = ±0.23, 6 data sets). (TIF) [file pone.0124070.s002.tif]

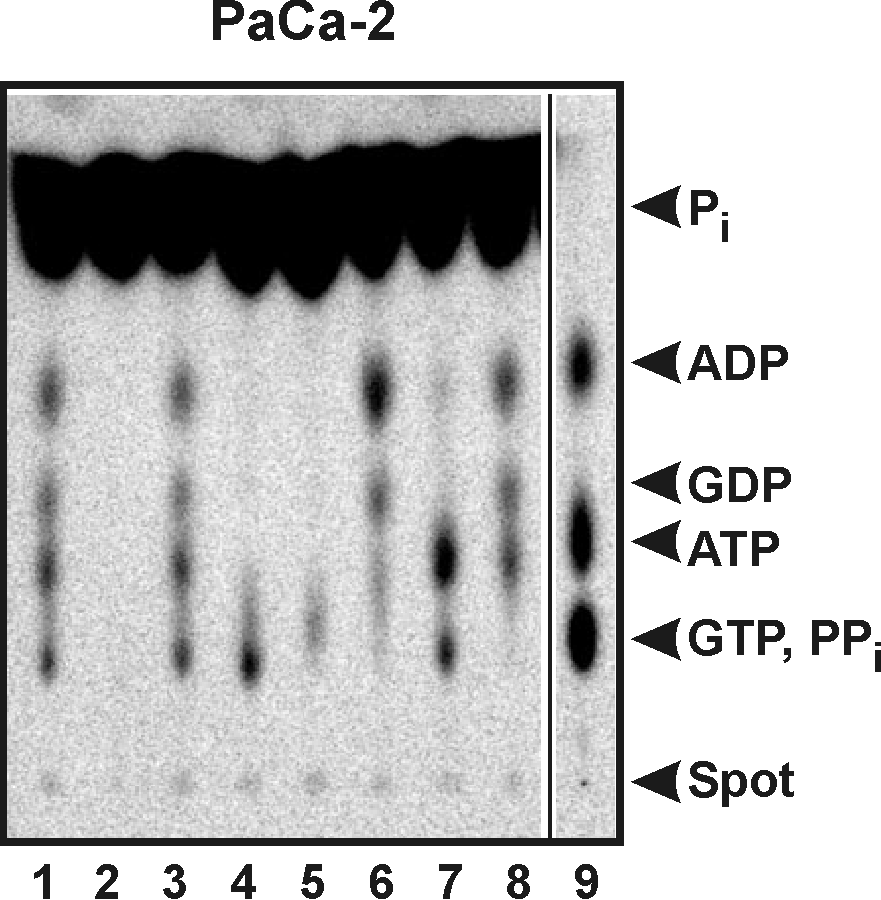

Supplement: S3 Fig — The products of acidification of 32Pi loaded PaCa-2 cells similar to Fig 3B were diluted with 4 volumes of buffer (10 mM tris pH 8.0, 0.2 mM EDTA and 1 mM MgCl2) and subjected to different enzymatic reactions that are characteristic of the nucleotides as discussed in the methods. This is then subjected to TLC analysis. The enzymes and the substrates used for each characteristic reaction and their effects are noted for each lane (spot). The appearance (or increased intensity) or disappearance (or reduced intensity) of the bands upon specific reactions confirmed their identity. Lanes 1, untreated; lane 2, phosphatase (P-ase): all the nucleotides disappeared; lane 3, pyrophosphatase (PPi-ase): no change was observed. Lane 4, phosphodiesterase (PDE): cleaves at the α-β phosphate bond—generated PPi from ATP and GTP which migrates at the same place as GTP and generated Pi from ADP and GDP; lane 5, treatment with PDE followed by PPi-ase: PPi produced from PDE gets hydrolyzed by PPi-ase and the PPi band disappeared—confirms that the product of PDE is PPi; lane 6, adenylate kinase (ADK) and excess AMP (1 mM): ATP diminished while ADP intensified; GTP, a weak substrate for ADK, also diminished; lane 7, nucleotide diphosphokinase (NDK) and excess ATP (1 mM): ADP and GDP diminished while ATP and GTP intensified; lane 8, NDK and excess ADP: GTP diminished; lane 9, 32P-(ADP, ATP, GTP) standard. 32PPi migrates at the same position as GTP. (TIF) [file pone.0124070.s003.tif]

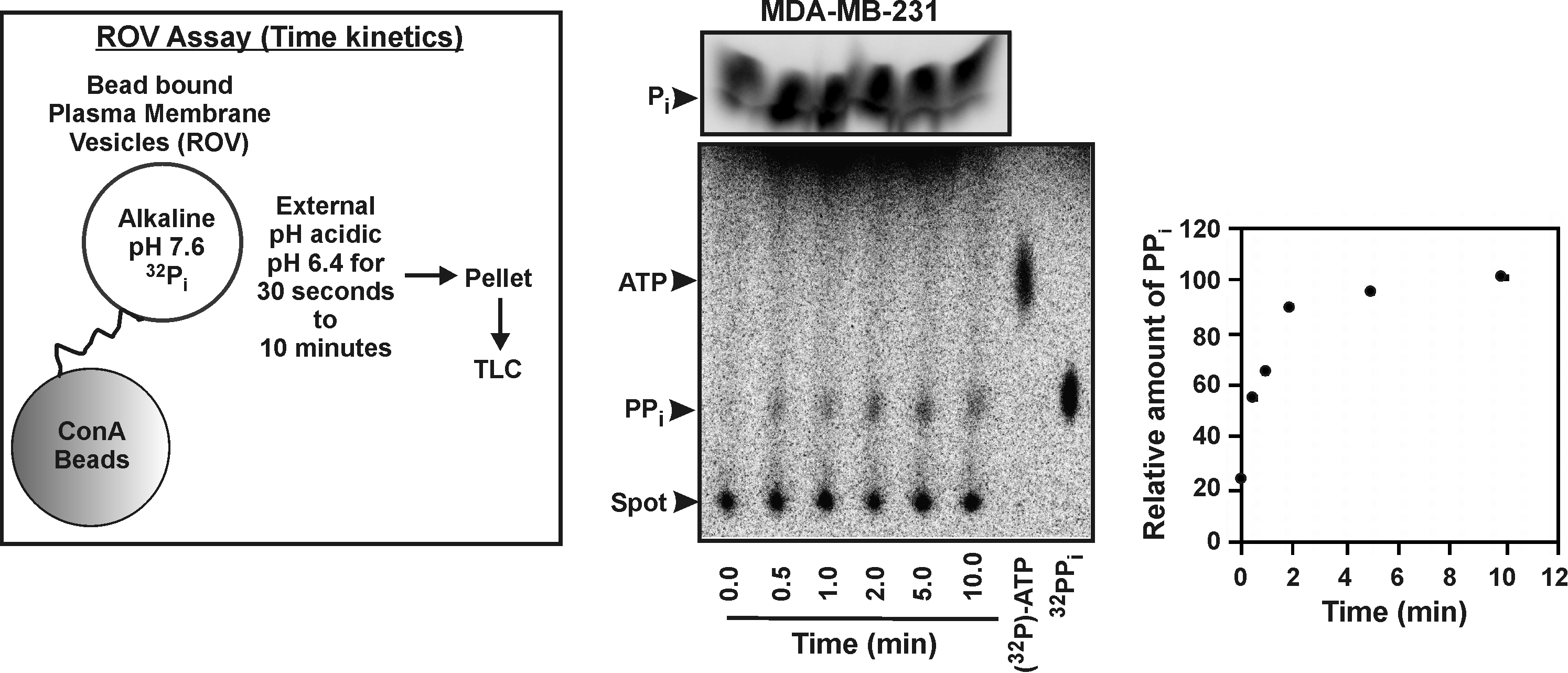

Supplement: S4 Fig — Suspensions of plasma membrane vesicles from MDA-MB-231 loaded with 32Pi at pH 7.6 and bound to conA beads were acidified to pH 6.4 for the indicated amount of time, processed and analyzed by TLC as shown in the schematic. Time interval between addition of acid and lysis by chloroform was taken as the time of acidification. The load controls for 32Pi in the TLC are shown above at a lower exposure. The amounts of PPi were corrected for the load using the Pi band intensity. Plot of relative amount of PPi is shown taking the highest value as 100. Full range time kinetics was done 2 times but kinetics with less number of time points was done 4 times. (TIF) [file pone.0124070.s004.tif]
